# Boosting Replication and Penetration of Oncolytic Adenovirus by Paclitaxel Eradicate Peritoneal Metastasis of Gastric Cancer

**DOI:** 10.1016/j.omto.2020.06.021

**Published:** 2020-06-25

**Authors:** Wataru Ishikawa, Satoru Kikuchi, Toshihiro Ogawa, Motoyasu Tabuchi, Hiroshi Tazawa, Shinji Kuroda, Kazuhiro Noma, Masahiko Nishizaki, Shunsuke Kagawa, Yasuo Urata, Toshiyoshi Fujiwara

**Affiliations:** 1 Department of Gastroenterological Surgery, Okayama University Graduate School of Medicine, Dentistry and Pharmaceutical Sciences, Okayama 700-8558, Japan; 2 Center for Innovative Clinical Medicine, Okayama University Hospital, Okayama 700-8558, Japan; 3 Oncolys BioPharma, Inc., Tokyo 106-0032, Japan

**Keywords:** peritoneal metastasis, adenovirus, gastric cancer, intraperitoneal chemotherapy, paclitaxel, oncolytic virus

## Abstract

Peritoneal metastasis is the most frequent form of distant metastasis and recurrence in gastric cancer, and the prognosis is extremely poor due to the resistance of systemic chemotherapy. Here, we demonstrate that intraperitoneal (i.p.) administration of a green fluorescence protein (GFP)-expressing attenuated adenovirus with oncolytic potency (OBP-401) synergistically suppressed the peritoneal metastasis of gastric cancer in combination with paclitaxel (PTX). OBP-401 synergistically suppressed the viability of human gastric cancer cells in combination with PTX. PTX enhanced the antitumor effect of OBP-401 due to enhanced viral replication in cancer cells. The combination therapy increased induction of mitotic catastrophe, resulting in accelerated autophagy and apoptosis. Peritoneally disseminated nodules were selectively visualized as GFP-positive spots by i.p. administration of OBP-401 in an orthotopic human gastric cancer peritoneal dissemination model. PTX enhanced the deep penetration of OBP-401 into the disseminated nodules. Moreover, a non-invasive *in vivo* imaging system demonstrated that the combination therapy of i.p. OBP-401 administration with PTX significantly inhibited growth of peritoneal metastatic tumors and the amount of malignant ascites. i.p. virotherapy with PTX may be a promising treatment strategy for the peritoneal metastasis of gastric cancer.

## Graphical Abstract

**Figure:**
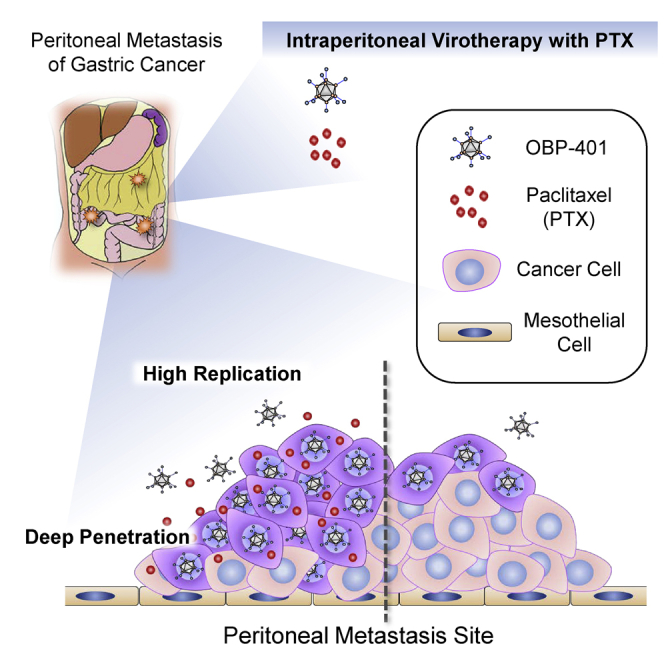

## Teaser

Paclitaxel (PTX) enhanced the replication of telomerase-specific oncolytic adenovirus in cancer cells and the penetration of the virus into the disseminated nodules of gastric cancer (GC) in the abdominal cavity. The intraperitoneal virotherapy combined with PTX may be a novel promising therapy for peritoneal metastasis of GC.

## Introduction

Peritoneal metastasis is the most common form of distant metastasis and recurrence in gastric cancer, and the prognosis remains extremely poor, even with current advanced modalities.^1^, ^2^, ^3^ At present, no curative treatment options exist for peritoneal metastasis. Systemic palliative chemotherapy is generally administered to patients with peritoneal metastasis.^4^^,^^5^ Systemic administration of anti-cancer drugs is limited for the treatment of peritoneal carcinomatosis because the peritoneal-plasma barrier prevents the delivery of anti-cancer drugs from the blood into the peritoneal cavity.^6^^,^^7^ Intraperitoneal (i.p.) administration of chemotherapeutic agents is advantageous for peritoneal carcinomatosis compared to systemic chemotherapy because anti-cancer drugs can be directly delivered into the peritoneal cavity, and high concentrations can be maintained in the peritoneal cavity. Several phase III trials have demonstrated that i.p. chemotherapy has a survival benefit compared with systemic chemotherapy in patients with advanced ovarian cancer.^8^, ^9^, ^10^ In gastric cancer with peritoneal metastasis, some phase II trials have demonstrated the effectiveness of i.p. paclitaxel (PTX), which can be retained in the peritoneal cavity for a long time due to its molecular characteristics including its large size and water insolubility, in addition to systemic chemotherapy.^11^, ^12^, ^13^ Recently, a phase III trial has suggested the possibility of improved survival of i.p. PTX with systemic chemotherapy.^14^ However, the outcome of gastric cancer patients with peritoneal metastasis is unsatisfactory, and new or improved strategies are needed.

Oncolytic viruses are designed to replicate selectively within tumor cells and lyse infected tumor cells.^15^ We have developed an attenuated adenovirus (Ad), OBP-301 (Telomelysin), which drives the E1A and E1B genes for viral replication under control of the human reverse transcriptase promoter, and we previously confirmed its antitumor effects in various human tumor cells including gastric cancer.^16^, ^17^, ^18^, ^19^ Furthermore, a phase I trial of OBP-301 for patients with various advanced solid tumors demonstrated that OBP-301 is well tolerated by patients.^20^ We have further developed OBP-401 (TelomeScan), which is a modified OBP-301, to express green fluorescent protein (GFP) to visualize viable cancer cells.^21^, ^22^, ^23^ OBP-401 enables detection of a few viable cancer cells as GFP-positive cells in the clinical peritoneal washes of advanced gastrointestinal cancer patients.^23^^,^^24^ Although systemic virotherapy is impractical due to the presence of neutralizing antibodies for Ad, locoregional administration such as to an intratumoral site or the i.p. cavity is a reasonable strategy.^25^

In this study, we show that i.p. administration of OBP-401 results in effective delivery to, and eradication of, peritoneal metastasis of gastric cancer. Furthermore, the antitumor activity of OBP-401 was markedly enhanced when used in combination with PTX, which enhanced the replication and penetration of OBP-401 in peritoneal metastasis of gastric cancer.

## Results

### *In Vitro* Synergistic Antitumor Effect of OBP-401 and PTX on Human Gastric Cancer Cells

**Figure 1:**
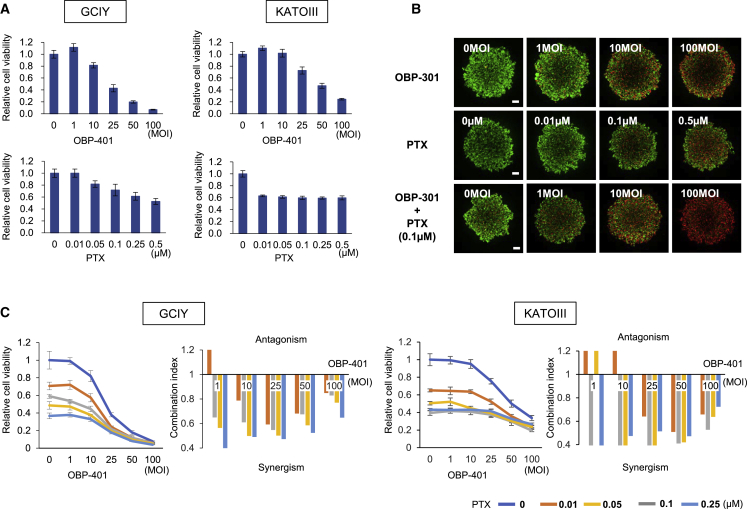
OBP-401 Synergistically Enhances the Antitumor Effect of PTX in Human Gastric Cancer Cells (A) GCIY and KATOIII cells were infected with OBP-401 at the indicated MOIs for 3 days. Cells were treated with PTX at the indicated doses for 24 h. Cell viability was quantified using the XTT assay. The cell viability of a mock-treated group was considered 1.0, and the relative cell viability was calculated. Data are expressed as the mean ± SD (n = 5). (B) For monotherapy, cells were infected with OBP-301 at the indicated MOIs for 72 h or treated with PTX at the indicated concentrations for 24 h. For combination therapy with OBP-301 and PTX, GCIY cells were infected with OBP-301 at the indicated MOIs. 2 days after viral infection, cells were treated with PTX (0.1 μmol/L) for 24 h. Cell viability and cytotoxicity were evaluated using the live and dead assay. Green color indicates live cells, and red color indicates dead cells. Scale bar, 200 μm. (C) The combination index was calculated with CalcuSyn software. Synergism and antagonism were defined as interaction indices of <1 and >1, respectively.

Previously, we developed an oncolytic Ad, OBP-401 (TelomeScan), which replicates selectively only within human cancer cells and expresses GFP.^21^^,^^23^ To evaluate the antitumor effect of OBP-401 and PTX, which is effective for peritoneal metastasis of gastric and ovarian cancer following i.p. administration,^8^, ^9^, ^10^, ^11^, ^12^, ^13^, ^14^ we treated GCIY or KATOIII cells with OBP-401 or PTX. Cell death was induced in both GCIY and KATOIII cells in a dose-dependent manner following treatment with OBP-401 or PTX (Figure 1A). We reported the chemosensitizing effect of OBP-301, which has the same basic structure as OBP-401 except for *GFP* (Figure S1), in several types of human malignant tumor cells.^26^, ^27^, ^28^, ^29^ To investigate the synergistic antitumor effect of OBP-401 and PTX in human gastric cancer cells, we evaluated the effect of combination therapy visually using a live and dead assay. We used OBP-301 instead of OBP-401 to distinguish live cells from dead cells in this experiment. The cytopathic effect of OBP-301 on human gastric cancer cells was equal to that of OBP-401 (Figure S2). Combination therapy suppressed the viability of GCIY and KATOIII cells more efficiently than monotherapy (Figure 1B; Figure S3). The sodium 3′-[1-(phenylaminocarbonyl)-3,4-tetrazolium]-bis(4-methoxy-6-nitro) benzene sulfonic acid hydrate (XTT) cell viability assay also demonstrated that combination therapy induced gastric cancer cell death in a dose-dependent manner. Calculation of the combination index indicated a synergistic antitumor effect of combination therapy in both types of human gastric cancer cells (Figure 1C). These results suggest that the combination of OBP-401 and PTX has a synergistic antitumor effect on human gastric cancer cells.

### Enhancement of Adenoviral Replication Efficiency in Human Gastric Cancer Cells by PTX

**Figure 2:**
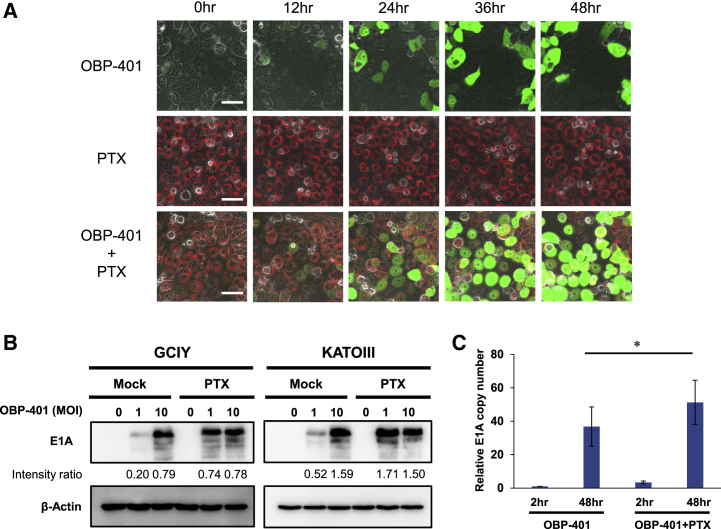
PTX Enhances the Replication Efficiency of OBP-401 in Human Gastric Cancer Cells (A) Time-lapse imaging of GCIY cells treated with OBP-401, PTX, or both. Green color indicates OBP-401 replication, and red color indicates PTX uptake. Scale bar, 50 μm. (B) Expression of adenoviral E1A proteins in GCIY and KATOIII cells treated with OBP-401 and PTX. β-actin was used as a loading control. The band intensity of E1A was normalized against that of β-actin and the ratios are shown. (C) Assessment of viral replication in GCIY cells. GCIY cells were treated with OBP-401 (1 MOI) or the combination of OBP-401 (1 MOI) and PTX (0.1 μM). Quantitative real-time PCR assay was performed to quantify the amount of viral E1A copy number. The copy number of viral E1A is defined as the E1A/GAPDH ratio relative to that of the sample 2 h after OBP-401 infection (2 h after OBP-401 infection = 1). Data are shown as means ± SD. Statistical significance was defined as p < 0.05 (single asterisk).

To investigate the mechanism underlying the synergistic antitumor effect of OBP-401 and PTX, we assessed whether PTX affects the replication of OBP-401 in human gastric cancer cells. For this purpose, GCIY cells treated with 10 nM PTX conjugated with red fluorescence were simultaneously infected with 10 multiplicity of infection (MOI) OBP-401, and the uptake of green and red fluorescence into the cancer cells was evaluated with time-lapse imaging. Red PTX fluorescence was immediately taken up into the cytoplasm of cancer cells. The replication of OBP-401 in the cancer cells was detected as GFP expression 24 h after virus administration. When combined with PTX, the number of GFP-expressing cells infected with OBP-401 was increased compared to monotherapy (Figure 2A; Video S1). Next, we performed western blot analysis and quantitative real-time PCR assay of viral E1A to assess whether PTX enhances the replication efficiency of OBP-401 in gastric cancer cells. Western blot analysis showed that the expression of adenoviral E1A was increased by the combination with PTX compared to monotherapy (Figure 2B). Moreover, quantitative PCR assay showed that the copy number of viral E1A was increased by the combination with PTX (Figure 2C). These results suggest that PTX enhances the replication efficiency of OBP-401 in human gastric cancer cells and synergistically enhances adenoviral oncolysis.

Video S1. Time-Lapse Imaging Spanning 48 h after Treatment Showed that PTX Enhanced the Replication of OBP-401 (GFP Expression) in GCIY Cells

### Increase in MC Induction by the Combination of PTX and Oncolytic Ad

**Figure 3:**
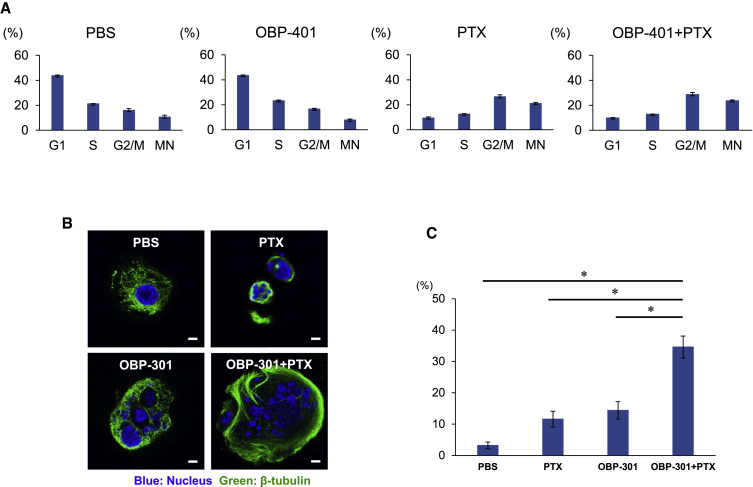
The Combination of Oncolytic Ad and PTX Increases Induction of Mitotic Catastrophe (MC) (A) The percentage of cells in each phase of the cell cycle was analyzed using FACS analysis in GCIY cells stained with propidium iodide after mock treatment or treatment with OBP-401, PTX, or the combination of OBP-401 and PTX. GCIY cells were infected with OBP-401 at a dose of 10 MOI. 2 days after OBP-401 infection, cells were treated with PTX at a dose of 0.1 μM for 24 h. The percentage of cells in G1, S, and G2/M and multi-nuclear cells were analyzed with FACS analysis. Data are expressed as mean values ± SD (n = 3). (B) Representative images of MC-associated nuclear morphology of GCIY cells 24 h after treatment with OBP-301, PTX, or both. Blue color indicates the nucleus, and green color indicates β-tubulin. Scale bar, 10 μm. (C) The percentage of MC was quantified from three different randomly selected fields under a confocal laser scanning microscope. Data are expressed as mean values ± SD. Statistical significance was defined as ∗p < 0.05.

To evaluate the underlying mechanism of the synergistic antitumor effect of oncolytic Ad and PTX, we analyzed the effects of combined treatment on cell-cycle progression. GCIY cells were infected with OBP-401, and 2 days after OBP-401 infection, cells were treated with PTX. 24 h after PTX treatment, fluorescence-activated cell sorting (FACS) analyses were performed. Monotherapy with PTX increased the number of cells in the G2/M phase and multi-nuclear cells, although 10 MOI of OBP-401 infection alone did not notably alter the cell-cycle profile (Figure 3A). Combination therapy with OBP-401 and PTX enhanced the accumulation of cells in the G2/M phase and multi-nuclear cells. Accordingly, we further analyzed the nuclear morphology of multi-nuclear cells because we expected that multi-nuclear cells were related to the synergistic effects of combination treatment of human gastric cancer cells.

Mitotic catastrophe (MC) is a distinctive process of cell death caused by abnormal mitosis.^30^ MC is characterized by unique morphological nuclear alterations such as multi-nucleation and/or micro-nucleation. 24 h after treatment, multi-nucleated giant nuclei with chromosome vesicles were observed in cells treated with PTX or OBP-301. When cells were treated with a combination of OBP-301 and PTX, giant multi-nucleated cells and micro-nucleated cells were observed (Figure 3B). Evaluation of MC development was performed by counting nuclei displaying morphological changes characteristic of MC. The combination of OBP-301 and PTX significantly increased MC induction in human gastric cancer cells (Figure 3C). This result suggests that MC is related to the synergistic antitumor effect of combination therapy for human gastric cancer cells.

### Enhancement of OBP-401-Induced Apoptosis and Autophagy by PTX

**Figure 4:**
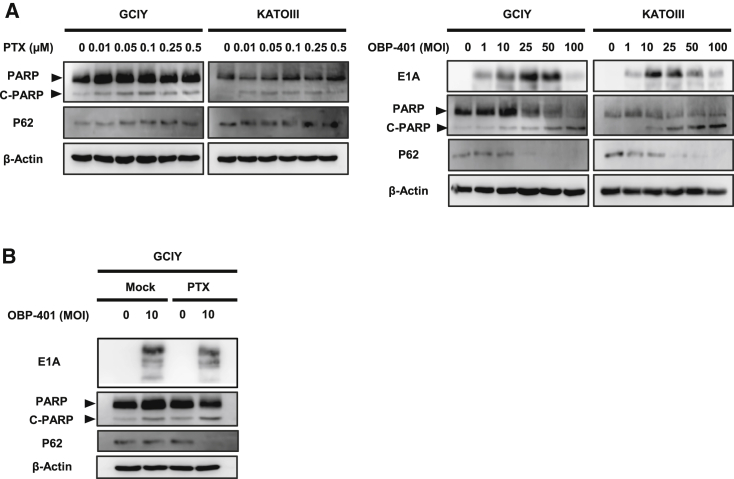
PTX Enhances OBP-401-Induced Apoptosis and Autophagy by Increasing MC (A) Expression of PARP, cleaved PARP (C-PARP), and p62 proteins in GCIY and KATOIII cells treated with PTX at the indicated doses. Expression of PARP, C-PARP, p62, and adenoviral E1A proteins in cells infected with OBP-401 at the indicated MOIs. (B) Expression of PARP, C-PARP, p62, and adenoviral E1A proteins in GCIY cells treated with OBP-401 and PTX at 0.1 μM. β-actin was used as a loading control.

To evaluate whether MC induction with combination therapy can prime or accelerate cancer cells to different types of cell death, we used western blot analysis to assess induction of apoptosis and autophagy after treatment. Western blot analysis was performed 72 h after OBP-401 infection and revealed that OBP-401 or PTX treatment increased apoptosis, as confirmed by the accumulation of cleaved poly (ADP-ribose) polymerase (PARP) in human gastric cancer cells. Assessment of p62 downregulation revealed that OBP-401 infection stimulated autophagy, although PTX treatment did not (Figure 4A). When combined with PTX, OBP-401 enhanced expression of cleaved PARP compared to monotherapy. Moreover, when combined with PTX, p62 was downregulated by a lower dose of OBP-401 compared to monotherapy (Figure 4B). These results suggest that apoptosis and autophagy were accelerated by combination therapy as a result of MC induction.

### Labeling Peritoneal Carcinomatosis with OBP-401 and Selective Replication in Disseminated Nodules

**Figure 5:**
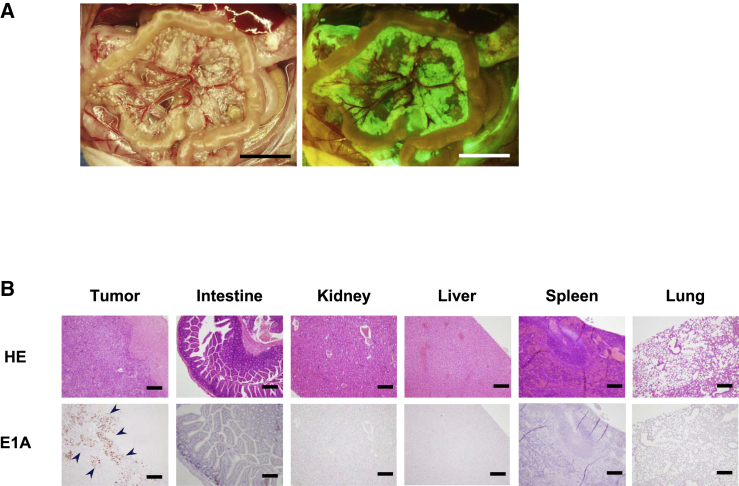
i.p. Injection of OBP-401 Allowed Selective Visualization of Peritoneal Dissemination of GCIY Cells and Replication (A) 4 weeks after inoculation of GCIY cells into the peritoneal cavity, 1 × 10^5^ PFU of OBP-401 was injected into the peritoneal cavity. 5 days later, disseminated nodules were visualized with GFP fluorescence. Scale bar, 1 mm. (B) Histological analysis of tissue sections of various organs. Upper panel, H&E staining. Scale bar, 200 μm. Lower panel, immunohistochemical staining for adenoviral E1A protein showing selective replication within peritoneal tumor cells. The nuclei were counterstained with hematoxylin. Positive staining is reddish brown (blue arrowheads). Scale bar, 200 μm.

To verify that OBP-401 injected into the abdominal cavity could infect peritoneal metastatic nodules and selectively replicate within them, we injected 500 μL solution containing OBP-401 into the abdominal cavity of nude mice bearing peritoneal carcinomatosis of human gastric cancer. 7 days after virus injection, disseminated nodules were detected as GFP fluorescence with fluorescence imaging (Figure 5A). Moreover, immunohistochemical staining revealed that the adenoviral E1A protein was selectively expressed in disseminated nodules, but not in surrounding visceral organs including liver (Figure 5B). These results suggest that i.p. injection of OBP-401 selectively infected and labeled peritoneal disseminated cancer.

### i.p. Virotherapy Combined with PTX for Peritoneal Carcinomatosis

**Figure 6:**
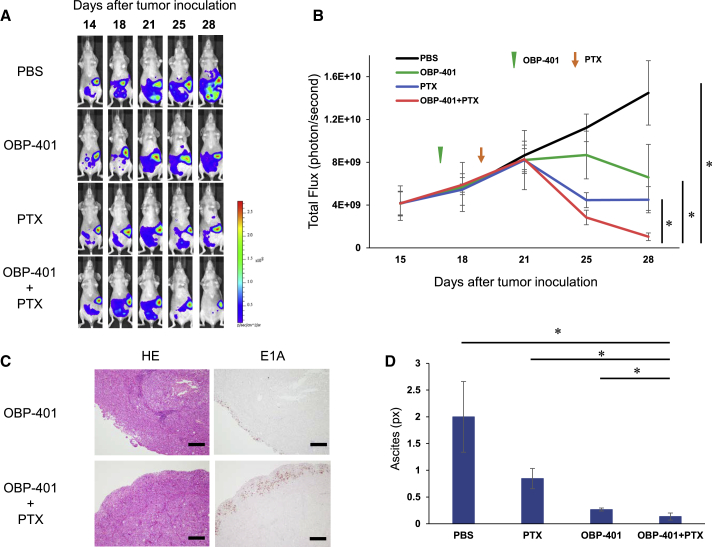
Enhancement of Antitumor Effects of i.p. Administration of OBP-401 in Combination with PTX in the Peritoneal Carcinomatosis Mouse Model of Gastric Cancer GCIY-Luc cells (5 × 10^6^ cells) were inoculated into the abdominal cavity of athymic nude mice. A 500 μL volume of solution containing OBP-401 (1 × 10^5^ PFU) was i.p. injected into the abdominal cavity at 17 days after tumor inoculation. PTX (10 mg/kg body weight) was also i.p. injected 2 days after OBP-401 injection. (A) Representative photographs of tumor-bearing mice treated with PBS, OBP-401, PTX, or OBP-401, and PTX. (B) The luminescence in tumor tissue was analyzed using the IVIS system at 15, 18, 21, 25, and 28 days after tumor inoculation. Data are expressed as mean values ± SD (n = 6). Statistical significance was defined as ∗p < 0.05. (C) Histological analysis of peritoneal nodules of GCIY tumors. Tumor tissues were obtained at 28 days after tumor inoculation. Left, H&E staining. Scale bar, 200 μm. Right, immunohistochemical staining for adenoviral E1A protein. Scale bar, 200 μm. (D) Peritoneal ascites were measured with computed tomography (CT) at 35 days after tumor inoculation. The amount of ascites was calculated at the fifth lumbar level on CT using ImageJ software. Data are expressed as the mean values ± SD (n = 4). Statistical significance was defined as ∗p < 0.05.

Finally, we examined whether i.p. administration of OBP-401 combined with PTX could eradicate peritoneal disseminated cancer using a GCIY-Luc xenograft mouse model, because KATOIII cells do not have tumorigenic ability. OBP-401 or PBS was i.p. injected 17 days after tumor inoculation. PTX was i.p. injected 2 days after OBP-401 injection for only one cycle. The combination of OBP-401 and PTX significantly suppressed i.p. tumor growth compared to PBS or monotherapy with OBP-401 or PTX (Figures 6A and 6B). Histopathological examination of resected peritoneal nodules showed that adenovirus in the combination therapy-treated tumors infiltrated more deeply into the tumor compared to OBP-401-treated tumors (Figure 6C; Figure S7). Moreover, 2 weeks after treatment, the amount of fluid in the abdominal cavity in the virotherapy groups was significantly decreased compared to PBS or PTX treatment (Figure 6D). These results suggest that i.p. virotherapy has therapeutic potential to eradicate peritoneal carcinomatosis of gastric cancer, and that PTX enhances the antitumor effect of OBP-401 for peritoneal metastasis.

## Discussion

At present, existing treatments for peritoneal dissemination of gastric cancer have not significantly improved clinical outcomes. In this study, we demonstrated the therapeutic potential of i.p. administration of OBP-401 in combination with PTX for peritoneal dissemination of gastric cancer. i.p. administration of antitumor agents is a reasonable and effective treatment strategy for peritoneal metastasis because these agents can directly reach i.p. cancer cells. In our previous studies, we performed intra-tumoral injection of OBP-301 into primary tumor sites and showed that the tumor-specific, replication-selective oncolytic Ad, OBP-301, has antitumor effects in various types of cancers.^16^^,^^18^, ^19^, ^20^^,^^31^ Moreover, we have shown that i.p. administration of OBP-301 could spread to the peritoneal cavity and infect viable cancer cells,^23^^,^^32^ resulting in antitumor effects on peritoneal metastasis.^28^

In preclinical studies, the efficacy of an oncolytic Ad combined with chemotherapeutic agents such as docetaxel, gemcitabine, Cisplatin (CDDP), doxorubicin, and PTX in several human cancer cells has been previously reported.^27^, ^28^, ^29^^,^^33^^,^^34^ OBP-301 and OBP-401 enhance the antitumor effects of chemotherapeutic agents. However, anti-DNA synthesis agents such as gemcitabine, CDDP, and doxorubicin may have deleterious effects on viral replication because adenoviral E1A directs the progression of host cells from the G1/G0 phase to the S phase to optimize viral DNA replication. On the other hand, PTX is an antimicrotubular agent that stabilizes microtubules by preventing depolymerization and arrests the cell cycle in the G2 and M phases,^35^^,^^36^ which induces apoptosis, but does not inhibit DNA synthesis of host cells. Therefore, PTX is suitable for combination use with oncolytic Ad.

The combination of an Ad-mediated agent and taxanes has synergistic or additive effects in several cancers.^27^^,^^33^^,^^37^^,^^38^ However, the mechanism of the combination effect of these agents is unclear. We demonstrated that OBP-401 induced cancer cell lysis *in vitro*, and its effect was markedly enhanced by the combination with PTX (Figure 1). Western blot analysis and time-lapse imaging showed that PTX enhanced the replication efficiency of OBP-401 in gastric cancer cells (Figure 2). However, with flow cytometry analysis, we observed no increase in coxsackie adenovirus receptor expression, which is the primary receptor for Ad, when gastric cancer cells were treated with PTX (data not shown). PTX enhances adenoviral particle assembly and release, which results in synergistic effects on cancer cells.^38^^,^^39^ PTX may help Ad assembly in a microtubule-dependent manner because PTX increases the binding ability of Ad capsids to microtubules.^40^

PTX induces cell-cycle arrest in the G2/M phase as we showed with flow cytometry analysis (Figure 3A). The combination with OBP-401 and PTX increased the percentage of cancer cells in the G2/M phase. This observation suggests that OBP-401 enhances the percentage of cancer cells that enter PTX-mediated G2/M arrest by compulsive cell-cycle mobilization and G2/M trapping. Recently, we also reported that oncolytic Ad virus enhances the antitumor effects of quiescent cancer stem-like cells by induction of cell-cycle reentry.^19^ Oncolytic Ad virus directly damages DNA and causes aberrant mitosis in cancer cells, which results in MC. Moreover, multi-nuclear cells were increased with combination therapy.

MC is a novel oncosuppressive mechanism that senses mitotic failure and results in an irreversible fate of death such as apoptosis, autophagy, and necrosis in cancer cells.^30^^,^^41^ MC is not a separate mode of cell death but a process that decides how the cell will die.^42^ Microtubule-targeting agents such as PTX are well-known antimitotic agents, which induce MC by microtubule polymerization.^43^ The characteristic morphological changes of MC were detected following PTX treatment (Figure 3B). OBP-301 also induced MC in human gastric cancer cells, although wild-type Ad and replication-deficient Ad did not (Figure S4). This result suggests that OBP-401 directly damages the DNA of host cancer cells and forces them into cycling, which results in induction of MC and enhances the sensitivity of cancer cells to PTX.

The safety and feasibility of intratumoral injection of OBP-301 in patients with various types of advanced solid tumors have already been confirmed in a phase I clinical trial.^20^ Furthermore, phase I/II clinical studies of OBP-301 in combination with radiation therapy for esophageal cancer patients showed preliminary efficacy with no dose-limiting toxicities. Some phase I trials of i.p. administration of oncolytic virus have been performed in patients with ovarian cancer,^44^, ^45^, ^46^ and their safety profiles were confirmed. The safety of i.p. administration of PTX has been also confirmed in phase III trial for gastric cancer patients with peritoneal metastasis.^14^ There was no effect of each treatment on the body weight of mice *in vivo* study (Figure S5). We and our collaborators have confirmed that i.p. administration of OBP-301 in combination with CDDP has therapeutic efficacy for peritoneal metastasis of ovarian cancer in a xenograft model. In this study, i.p. administration of OBP-401 allowed selective visualization of disseminated nodules, and viral E1A protein was not detected in any other visceral organs (Figure 5), which indicates that i.p. virotherapy using OBP-401 has minimal adverse effects on normal i.p. organs. Furthermore, a dose-titration study (Figure S6) indicated that OBP-401 at 10^5^ plaque-forming units (PFUs), which is 3 logs lower than the concentration used for intratumoral injection, could still effectively suppress peritoneal dissemination, suggesting that i.p. administration of OBP-401 may easily lead to virus dispersion over the abdominal cavity and effective attachment to the disseminated nodules. Interestingly, PTX enhanced the penetration of OBP-401 into the disseminated nodules compared to OBP-401 alone (Figure 6C). One possible explanation for the deep penetration of OBP-401 by PTX may be that PTX enhanced the replication ability of OBP-401 in the disseminated nodules as we showed *in vitro*. Another possible reason is that OBP-401 may easily infiltrate into the peritoneal nodules because PTX decreases stromal fibrosis in human peritoneal mesothelial cells.^47^ Several times of treatment might cure the peritoneal metastasis of gastric cancer although only one cycle of treatment could not. Recently, we have reported that telomerase-specific oncolytic Ad virus such as OBP-301 could induce immunogenic cell death (ICD), which leads to the induction of systemic anti-tumor immune response via the activation of dendritic cells and the facilitation of cytotoxic T lymphocyte recruitment.^48^ Therefore, i.p. administration of oncolytic Ad virus treatment might be a promising candidate for combination therapy with immune checkpoint inhibitors (ICIs) in the future. Indeed, i.p. administration of OBP-401 in combination with PTX significantly decreased malignant ascites, which indicates that this novel treatment may not only improve the survival of patients with peritoneal metastasis but also maintain the quality of life of patients.

In conclusion, we demonstrated that i.p. administration of the telomerase-specific, replication-selective Ad, OBP-401, in combination with PTX efficiently eradicated peritoneal dissemination of gastric cancer in an orthotopic xenograft model. Moreover, the combination of OBP-401 and PTX strongly enhanced MC induction due to compulsive cell-cycle mobilization of OBP-401. Future clinical trials of i.p. virotherapy in combination with PTX for peritoneal metastasis are warranted to investigate the efficacy and tolerability.

## Materials and Methods

### Cell Lines

Two human gastric cancer cell lines were used in this study. GCIY cells were obtained from RIKEN Cell Bank (Tsukuba, Japan), and KATOIII cells were obtained from Health Science Research Resources Bank (Osaka, Japan). GCIY cells were maintained in Eagle’s minimum essential medium supplemented with 15% heat-inactivated fetal bovine serum (FBS; Sigma-Aldrich, St. Louis, MO, USA). KATOIII cells were maintained in a 1:1 mixture of Eagle’s minimum essential medium and RPMI 1640 supplemented with 10% FBS. GCIY cells transfected with the firefly luciferase plasmid vector (GCIY-Luc) were maintained in Eagle’s minimum essential medium supplemented with 15% FBS containing 0.2 mg/mL Geneticin (G418; Invitrogen, Carlsbad, CA, USA). All media were supplemented with 100 U/mL penicillin and 100 mg/mL streptomycin. The cells were routinely maintained at 37°C in a humidified atmosphere with 5% CO_2_.

### Recombinant Ad and Chemotherapeutic Agent

The recombinant, telomerase-specific, replication-competent Ad vector OBP-301 (Telomelysin) was previously constructed and characterized.^16^, ^17^, ^18^ OBP-401 (TelomeScan) is a telomerase-specific, replication-competent Ad variant in which the replication cassette and *GFP* under control of the cytomegalovirus promoter were inserted into the E3 region for monitoring of viral replication.^21^^,^^49^ The E1A-deleted Ad vector lacking a cDNA insert (dl312) and wild-type Ad (OBP-202) were also used as control vectors. Viruses were purified by ultracentrifugation using CsCl step gradients. Viral titers were determined by a plaque-forming assay using 293 cells, and the virus was stored at −80°C. PTX was purchased from Nippon Kayaku (Tokyo, Japan) and dissolved in PBS.

### Cell Viability Assay

GCIY and KATOIII cells were seeded on 96-well plates at a density of 1 × 10^3^ cells/well and cultured for 24 or 72 h before viral infection or administration of PTX. For monotherapy, cells were infected with OBP-401 at MOIs of 0, 1, 10, 25, 50, or 100 PFUs/cell for 72 h, or treated with PTX at a concentration of 0, 0.01, 0.05, 0.1, 0.25, or 0.5 μmol/L for 24 h. For combination therapy with OBP-401 and PTX, 48 h after viral infection with OBP-401 at the indicated MOIs, cells were further treated with PTX for 24 h. Cell viability was examined by using the Cell Proliferation Kit II (Roche Molecular Biochemicals, Indianapolis, IN, USA), which is based on a sodium 3′-[1-(phenylaminocarbonyl)-3,4-tetrazolium]-bis(4-methoxy-6-nitro) benzene sulfonic acid hydrate (XTT) assay, according to the manufacturer’s protocol. The combination index was calculated with CalcuSyn software (BioSoft, Cambridge, UK). Computation of the combination index was based on the methods of Chou and Talalay.^50^

### Live and Dead Assay

Cells were seeded on an ultra-low attachment, round-bottom 96-well plate (Corning Costar, Sigma-Aldrich) at a density of 5 × 10^3^ cells/well and cultured for 24 or 72 h before viral infection or administration of PTX. For monotherapy, cells were infected with OBP-301 at the indicated MOIs for 72 h or treated with PTX at the indicated concentrations for 24 h. For combination therapy, after 48 h of viral infection, cells were further treated with PTX (0.1 μmol/L). After 24 h of PTX administration, cell viability and cytotoxicity were evaluated with a solution of Calcein (4 μM) and EthD-1(4 μM) dissolved in PBS. Cells that formed spheroids were imaged using a confocal laser scanning microscope (LSM780, Zeiss, Jena, Germany).

### Evaluation of MC

Cells were seeded for 72 h on a chamber slide (Thermo Fisher Scientific, Waltham, MA, USA) and conjugated with OBP-301, PTX, or both for 24 h. After each treatment, cells were fixed for 15 min in 4% formaldehyde at room temperature. Protein blocking was done with 5% bovine serum albumin-PBS for 10 min at room temperature followed by incubation with β-tubulin antibody (Cell Signaling Technology, Danvers, MA, USA) for 24 h at 4°C. Alexa Fluor 488 anti-rabbit secondary antibody (Thermo Fisher Scientific) was then applied for 60 min at room temperature. Nuclear morphology was counterstained by using VECTASHIELD Hardset Antifade Mounting Medium with DAPI (Vector Laboratories, Burlingame, CA, USA). The morphological changes of the nuclei and cytoskeleton were examined under a LSM780 confocal laser scanning microscope (Zeiss). Images were processed with Imaris 7.6 (Bitplane, Belfast, UK).

### Cell-Cycle Analysis

GCIY cells were seeded on six-well plates at a density of 10 × 10^4^ cells/well and cultured for 24 h. After culturing for 24 h, cells were infected with OBP-401 (10 MOI), and after 48 h of viral infection, cells were further treated with PTX (0.1 μM). After 24 h of PTX administration, cells were resuspended and analyzed with FACScan (BD FACSLyric, BD Biosciences, Franklin Lakes, NJ, USA). The fluorescence-activated cell sorting (FACS) data were further analyzed using FlowJo Software (BD Biosciences) to calculate each fraction of cells in G1, S, and G2/M phases.

### Western Blot Analysis

GCIY and KATOIII cells were seeded in a 100-mm dish at a density of 1 × 10^5^ cells/dish and infected with OBP-401 at the indicated MOIs for 72 h. Cells were treated with PTX at the indicated doses for 24 h. Whole-cell lysates were prepared with lysis buffer (50 mM Tris-HCl [pH 7.4], 150 mM NaCl, 1% Triton X-100) containing a protease inhibitor cocktail (Complete Mini; Roche Applied Science, Mannheim, Germany). Proteins were electrophoresed on 8%–15% sodium dodecyl sulfate polyacrylamide gels and transferred to polyvinylidene difluoride membranes (Hybond-P; GE Healthcare, Buckinghamshire, UK). The membranes were blocked with Blocking-One (Nacalai Tesque, Kyoto, Japan) at room temperature for 30 min. The primary antibodies used were as follows: mouse anti-Ad5 E1A monoclonal antibody (BD PharMingen, Franklin Lakes, NJ, USA), rabbit anti-PARP polyclonal antibody, mouse anti-p62 antibody, and mouse anti-β-actin monoclonal antibody (Sigma-Aldrich). The secondary antibodies used were as follows: horseradish peroxidase-conjugated antibodies against rabbit IgG (GE Healthcare), mouse IgG (GE Healthcare), or goat IgG (Chemicon International, Temecula, CA, USA). Immunoreactive bands on the blots were visualized using enhanced chemiluminescence substrates (ECL Plus; GE Healthcare). The band intensities of E1A were analyzed by ImageJ software (NIH, Bethesda, MD, USA).

### Time-Lapse Imaging

Cells were seeded in a 27-mm glass base dish at a density of 3 × 10^4^ cells/well and cultured for 7 days. 7 days after cell seeding, cells were treated with OBP-401 (1 MOI) and PTX (0.1 μM), which was conjugated with red fluorescence (P7501; BODIPY FL paclitaxel, Thermo Fisher Scientific). Time-lapse images were taken serially at hourly intervals for 48 h after treatment using a confocal laser scanning biological microscope with a built-in culture incubator (FV-10i, Olympus, Tokyo, Japan).

### Quantitative Real-Time PCR Analysis

GCIY cells were treated with OBP-401 (1MOI) or the combination of OBP-401 (1 MOI) and PTX (0.1 μM). Total RNA (2 h and 48 h after treatment) was isolated from cells using the RNeasy Mini kits (QIAGEN, Hilden, Germany) according to the manufacturer’s instructions. The cDNA was synthesized from 1.0 μg of total RNA using Advantage RT PCR-kit (Clontech Laboratories, Mountain View, CA, USA). The quantitative real-time PCR was performed for gene expression analysis using the StepOnePlus Real-Time PCR System (Applied Biosystems, Waltham, MA, USA) with Taqman PCR master mix (Applied Biosystems, Foster City, CA, USA). The primers used in this study were: GAPDH (Applied Biosystems) and E1A (E1A-F: 5′-CCTGAGACGCCCGACATC-3′, E1A-R: 5′-GGACCGGAGTCACAGCTATCC-3′). GAPDH was used as a normalization control. The relative expression of each mRNA was determined using the ΔΔCt method.

### Animal Experiments

Animal experimental protocols were approved by the Ethics Review Committee for Animal Experimentation of Okayama University School of Medicine (number OKU-2015275). GCIY-Luc cells (5 × 10^6^ cells) were inoculated into the abdominal cavity of 6-week-old female BALB/c nu/nu mice (CLEA Japan, Tokyo, Japan). 17 days after cell inoculation, 500 μL solution containing OBP-401 (1 × 10^5^ PFU) or PBS was i.p. injected once. PTX (10 mg/kg body weight) was also i.p. injected 2 days after OBP-401 injection. Six mice were used for each group. To monitor tumor progression, we i.p. injected the substrate luciferin (Vivo Glo Luciferin; Promega, Madison, WI, USA) at a dose of 200 mg/kg body weight. Images were collected in the supine position every few min after luciferin injection with the Xenogen *in vivo* imaging system (IVIS) Lumina Imaging System (Caliper Life Sciences, Cheshire, UK), and photons emitted from the abdominal cavity were quantified by using Xenogen Living Image Software (Caliper Life Science). Peritoneal ascites and metastasis were measured by computed tomography (CT) 35 days after cancer cell inoculation. CT scanning was performed by 60-slice CT (Latheta LCT-200, HITACHI, Tokyo, Japan). The amount of ascites was measured at the fifth lumbar level on CT using ImageJ software. To monitor the replication of OBP-401 in the peritoneal cavity, we took *in vivo* fluorescence images at laparotomy using an Olympus SZX16 microscope and a DP71 camera (Olympus).

### Immunohistochemistry

For histological studies, peritoneal tumor nodules and visceral organs were removed and fixed in 10% neutralized formalin. All tissues were subsequently dehydrated in alcohol, embedded in paraffin, and sectioned for hematoxylin and eosin staining and immunohistochemical examination. After deparaffinization and rehydration, antigen retrieval was performed by microwave irradiation in 10 mM citrate buffer (pH 6.0). Following quenching of endogenous tissue peroxidases, tissue sections were incubated with rabbit anti-E1A polyclonal antibody (Santa Cruz Biotechnology, Dallas, TX, USA) or rabbit anti-adenovirus mAb (Abcam, Cambridge, MA, USA) to detect proliferating viral particles. Immunoreactive signals were visualized with a 3,39-diaminobenzidine tetrahydrochloride solution, and the nuclei were counterstained with hematoxylin. Sections were viewed under a microscope (BX50; Olympus).

### Statistical Analysis

We used the Student’s t test to identify statistically significant differences between groups. All data are expressed as means ± standard deviation (SD). p values less than 0.05 were considered statistically significant.

## Author Contributions

S. Kikuchi and T.F. developed the concept and designed research; W.I., T.O., M.T., and S. Kikuchi performed research and acquired data; W.I., S. Kikuchi, H.T., S. Kuroda, K.N., M.N., S. Kagawa, and T.F. analyzed and interpreted data; Y.U. supplied materials; W.I., S. Kikuchi, and T.F. wrote and reviewed the manuscript.

## Conflicts of Interest

Y.U. is the president and CEO of Oncolys BioPharma, the manufacturer of OBP-401 (TelomeScan). H.T. and T.F. are consultants for Oncolys BioPharma. The other authors declare no competing interests.

## Supplemental Information

Document S1. Figures S1–S7

Document S2. Article plus Supplemental Information
