# Supplementary material for: Boosting Replication and Penetration of Oncolytic Adenovirus by Paclitaxel Eradicate Peritoneal Metastasis of Gastric Cancer
Source: Mol Ther Oncolytics. 2020 Jun 25;18:262–71. doi: 10.1016/j.omto.2020.06.021 (PMC7378855; doi:10.1016/j.omto.2020.06.021)
Supplement: Document S1. Figures S1–S7 [file mmc1.pdf]

OMTO, Volume *18*

Supplemental Information

Boosting Replication and Penetration of Oncolytic Adenovirus by Paclitaxel Eradicate Peritoneal Metastasis of Gastric Cancer

Wataru Ishikawa, Satoru Kikuchi, Toshihiro Ogawa, Motoyasu Tabuchi, Hiroshi Tazawa, Shinji Kuroda, Kazuhiro Noma, Masahiko Nishizaki, Shunsuke Kagawa, Yasuo Urata, and Toshiyoshi Fujiwara

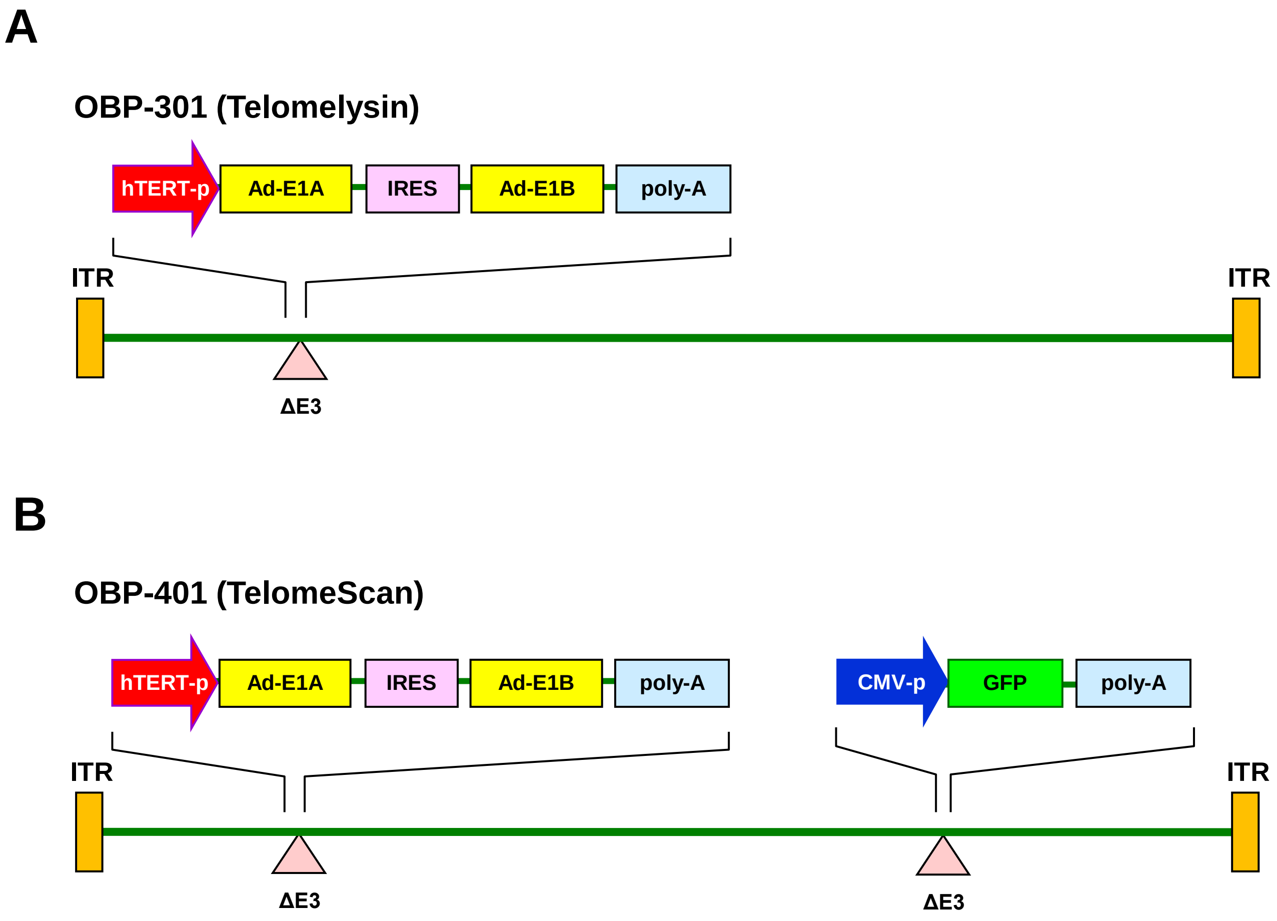

Supplementary Fig. S1

Schematic DNA structures of the telomerase-specific oncolytic adenoviruses. **A,** OBP-301 is a telomerase-specific replication-competent adenovirus that the hTERT promoter drives the expression of *E1A* and *E1B* genes linked by an internal ribosome entry site. **B,** OBP-401 is a variant of OBP-301in which the *GFP* gene is inserted into the E3 region, under the control of cytomegalovirus promoter for monitoring of viral replication.

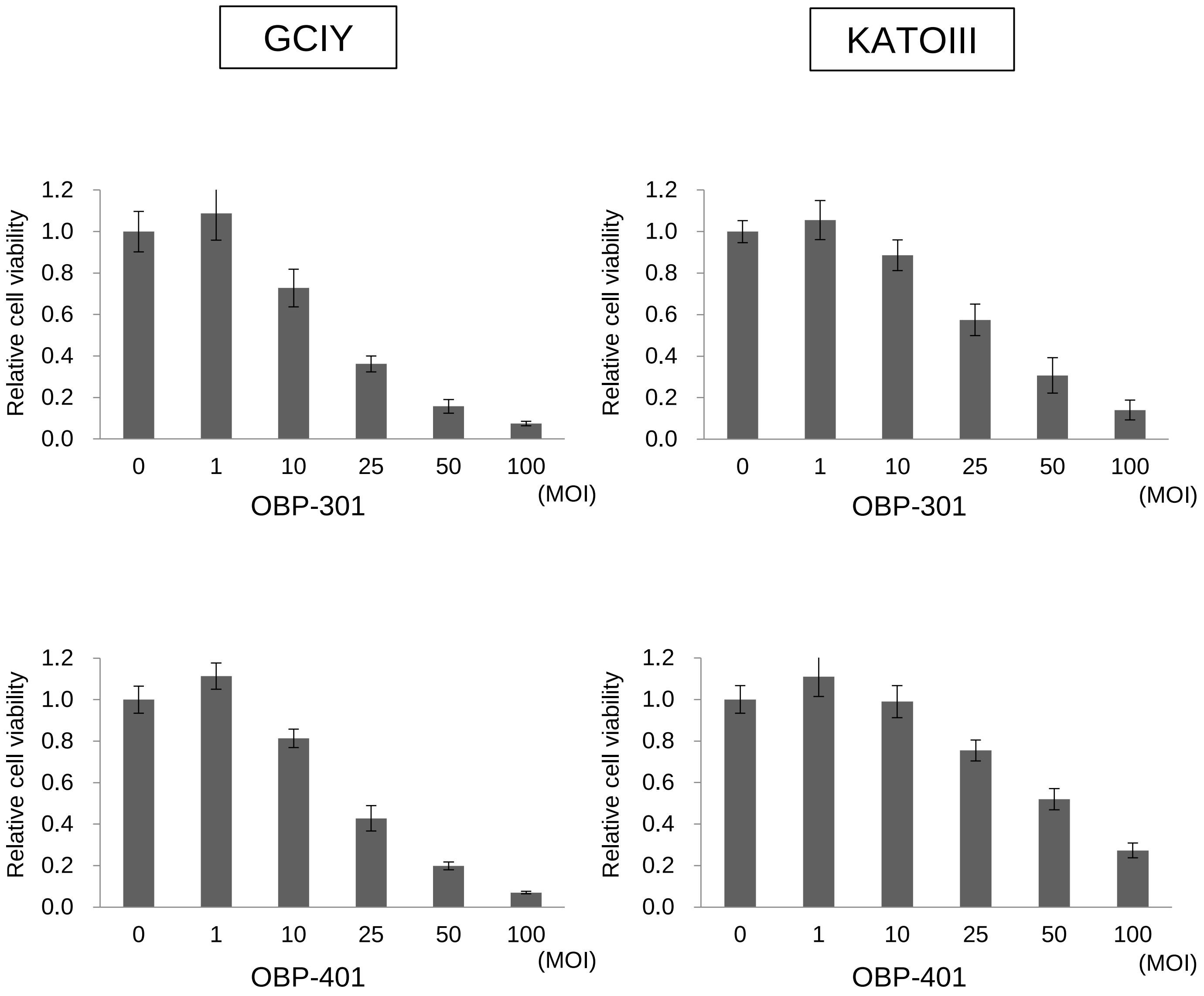

Supplementary Fig. S2

In vitro cytopathic effect of OBP-301 or OBP-401 on GCIY and KATOIII human gastric cancer cells. Cells were infected with OBP-301 or OBP-401 at the indicated MOIs for 3 days. Cell viability was quantified using XTT assay. The cell viability of a mock-treated group was considered 1.0 and the relative cell viability was calculated. Data are expressed as mean $\pm$ SD (n=5).

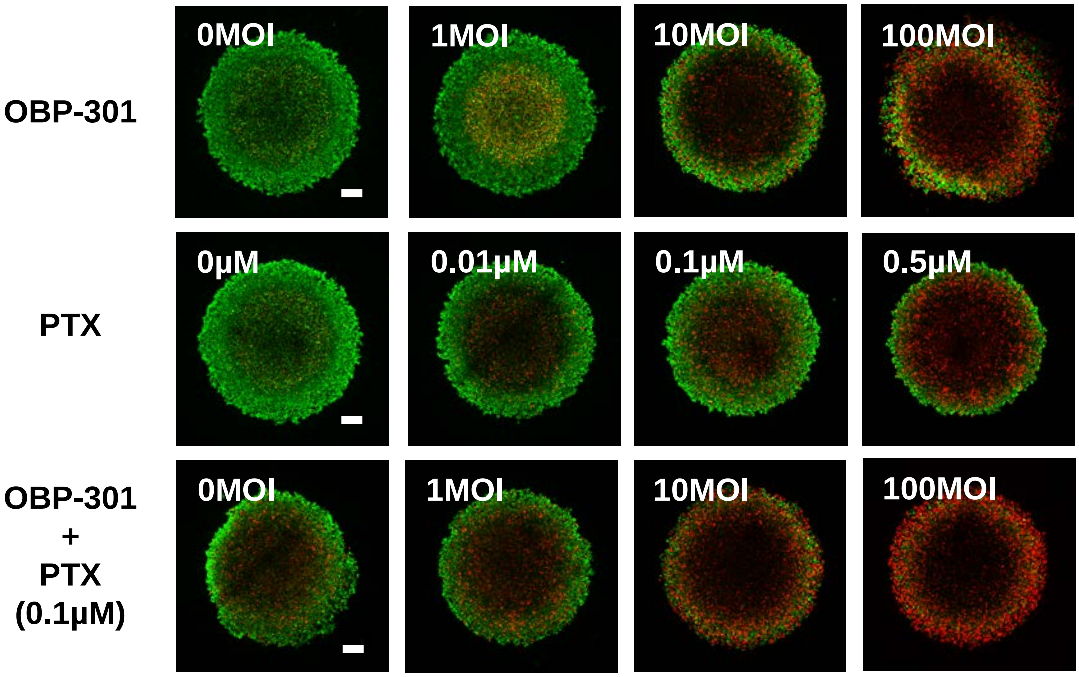

Supplementary Fig. S3

For monotherapy, cells were infected with OBP-301 at the indicated MOIs for 72 hours or treated with PTX at the indicated concentrations for 24 hours. For combination therapy with OBP-301 and PTX, KATOIII cells were infected with OBP-301 at the indicated MOIs. Two days after viral infection, cells were treated with PTX (0.1 μmol/l) for 24 hours. Cell viability and cytotoxicity were evaluated using the Live and Dead assay. Green color indicates live cells, and red color indicates dead cells. Scale bar, 200 μm.

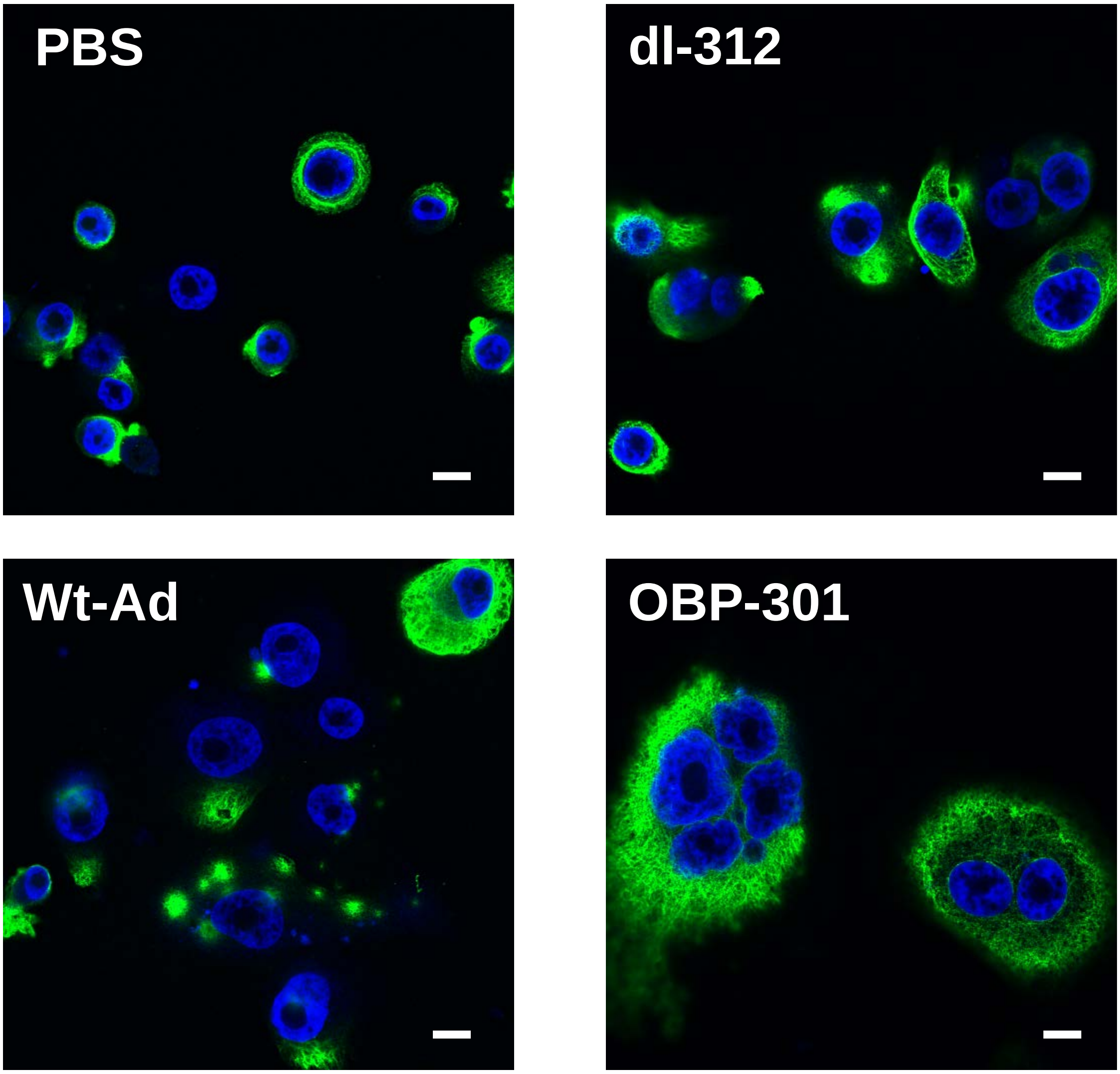

Supplementary Fig. S4

Appearance of Mitotic catastrophe (MC) change by OBP-301 infection. GCIY cells were infected with wild type Ad (Wt-Ad), replication-deficient Ad (dl-312) and OBP-301. Representative images of GCIY cells 24 hours after Ad infection. Blue color indicates nucleus and green color indicates β-tubulin. Scale bar, 10μm.

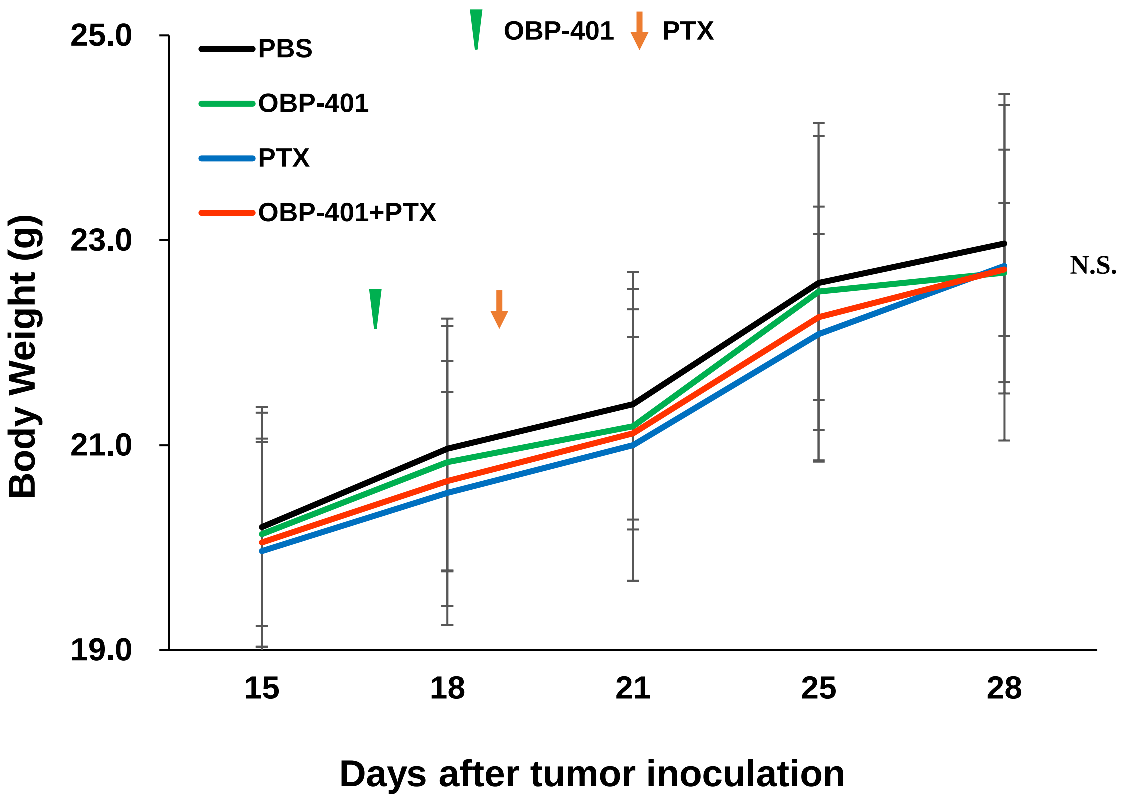

Supplementary Fig. S5

Peritoneal tumors of GCIY-Luc were treated with one time of intraperitoneal administration of OBP-401 followed by intraperitoneal administration of paclitaxel. The body weight of mice was monitored and plotted. Data are expressed as mean values ± SD (n=5). Statistical significance was defined as *P<0.05.

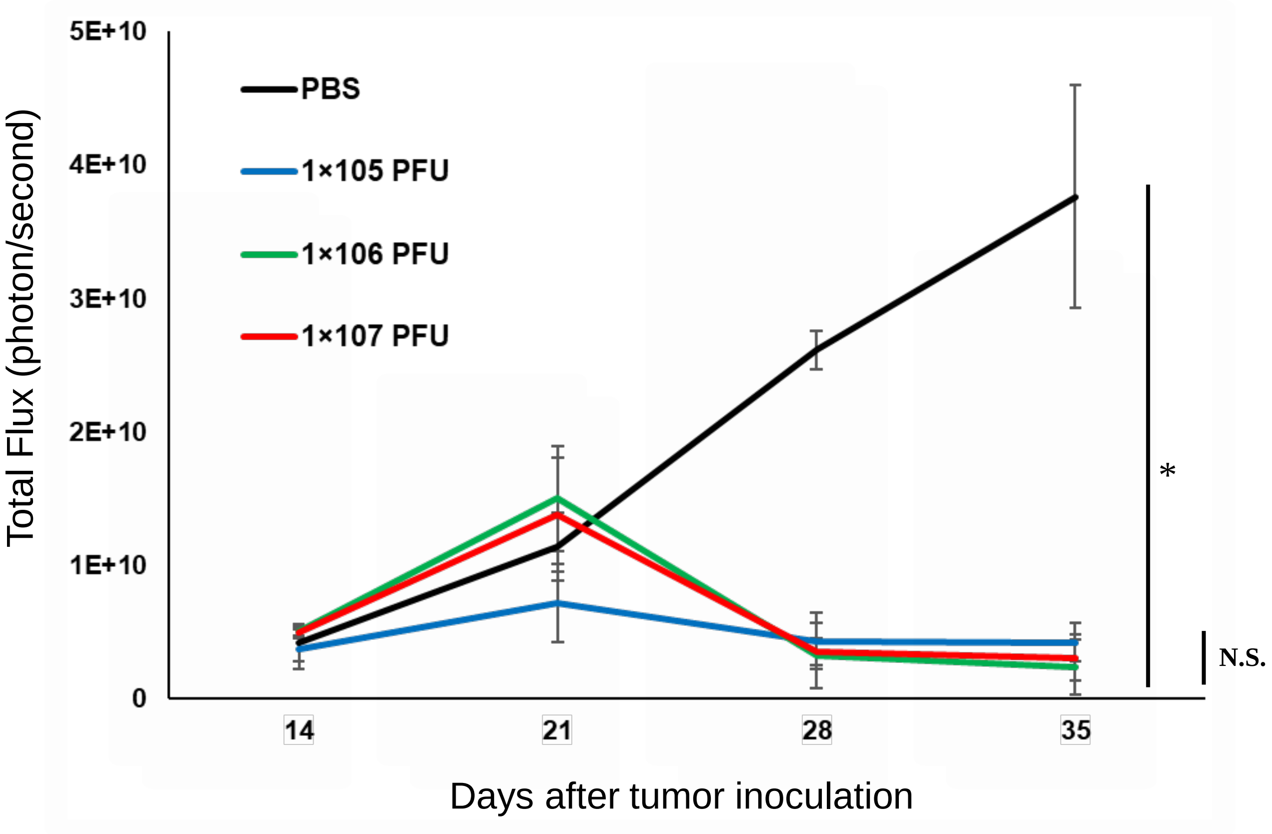

Supplementary Fig. S6

A dose-dependent antitumor effect of i.p. administration of OBP-401 to peritoneal carcinomatosis. Mice with peritoneal nodules of GCIY-Luc cells received i.p. administration of OBP-401 at the indicated MOIs on day 17. The luminescence in tumor tissue was analyzed using IVIS system on 14, 21, 28 and 35 days after tumor inoculation. Data are expressed as mean values ± SD (n=3). Statistical significance was defined as *P<0.05.

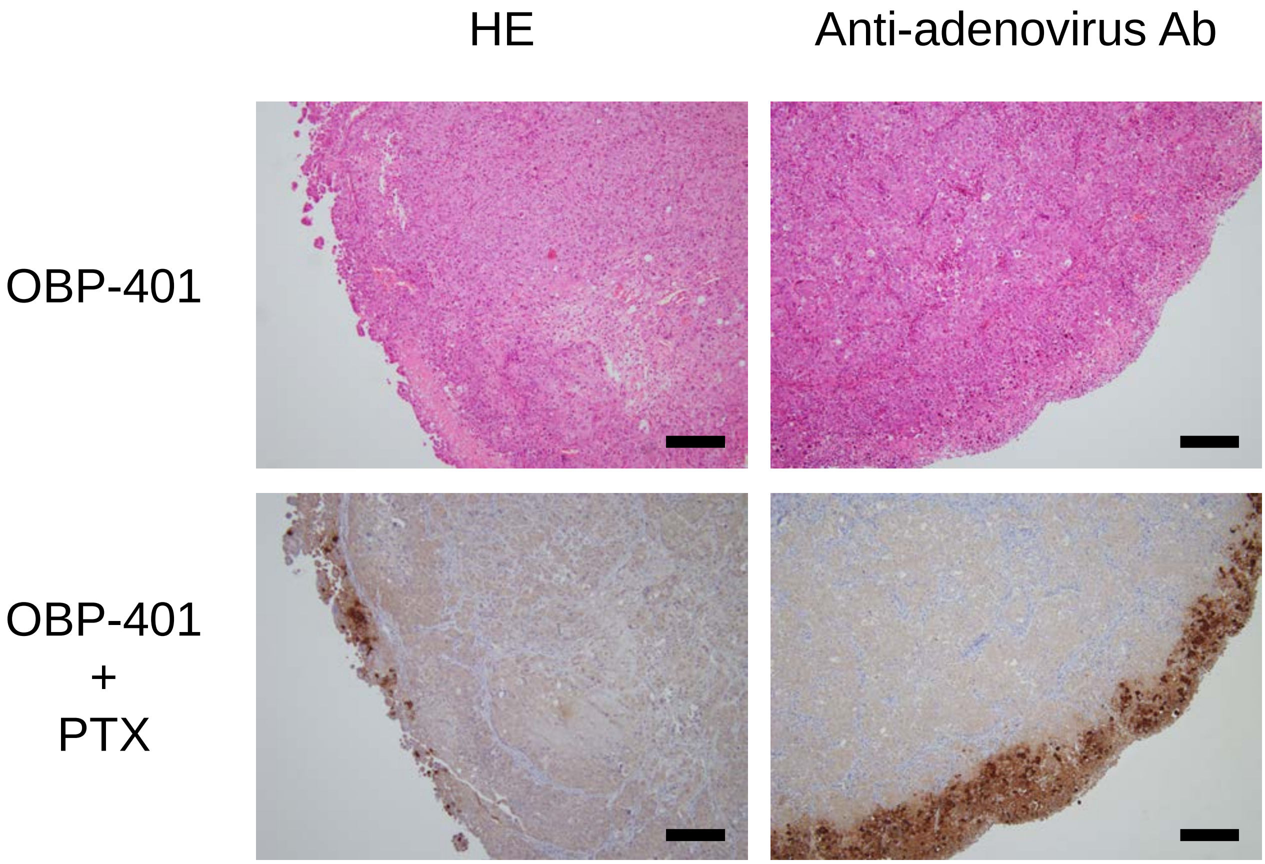

Supplementary Fig. S7

Histological analysis of peritoneal nodules of GCIY tumors. Tumor tissues were obtained at 28 days after tumor inoculation. Left, H&E staining. Scale bar, 200 µm. Right, immunohistochemical staining for anti-adenovirus mAb. Scale bar, 200 µm.
